# Combined Dietary Supplementation of *Tenebrio molitor* Larvae and Chitosan in Growing Pigs: A Pilot Study

**DOI:** 10.3390/vetsci11020073

**Published:** 2024-02-06

**Authors:** Christos Zacharis, Eleftherios Bonos, Chrysoula (Chrysa) Voidarou, Georgios Magklaras, Konstantina Fotou, Ilias Giannenas, Ioannis Giavasis, Chrysanthi Mitsagga, Christos Athanassiou, Efthimia Antonopoulou, Katerina Grigoriadou, Athina Tzora, Ioannis Skoufos

**Affiliations:** 1 Department of Agriculture, University of Ioannina, Kostakioi Artas, 47100 Arta, Greece; x.zaxaris@uoi.gr (C.Z.); ebonos@uoi.gr (E.B.); xvoidarou@uoi.gr (C.V.); gmag@uoi.gr (G.M.); kfotou@uoi.gr (K.F.); tzora@uoi.gr (A.T.); jskoufos@uoi.gr (I.S.); 2 School of Veterinary Medicine, Aristotle University of Thessaloniki, 54124 Thessaloniki, Greece; 3 Department of Food Science and Nutrition, School of Agricultural Sciences, University of Thessaly, 43100 Karditsa, Greece; igiavasis@uth.gr (I.G.); cmitsanga@uth.gr (C.M.); 4 Department of Agriculture, Plant Production and Rural Environment, University of Thessaly, 38446 Nea Ionia, Greece; athanassiou@uth.gr; 5 Department of Zoology, School of Biology, Aristotle University of Thessaloniki, 54124 Thessaloniki, Greece; eantono@bio.auth.gr; 6 Institute of Plant Breeding and Genetic Resources, Hellenic Agricultural Organization (ELGO)-DIMITRA, 57001 Thessaloniki, Greece; katgrigoriadou@elgo.gr

**Keywords:** insects, insect meal, chitin, swine, performance, health, gastrointestinal microbiota, meat quality

## Abstract

**Simple Summary:**

Currently, the worldwide livestock sector is facing the problem of producing sufficient feed resources. Alternative sources of proteinaceous ingredients are needed to feed the ever-increasing number of farm animals. Consumers are also aware of the negative environmental impacts of soybean and fishmeal production. For these reasons, novel feeding ingredients causing low environmental burden should be developed and used in the farm animal industries. In the present study, a combined dietary supplementation with *Tenebrio molitor* larvae and chitosan in pig diets was carried out. Forty-eight weaned pigs (34 days of life; mixed sex) were assigned into four treatment groups: a control group (Group A), a group supplemented with 10% *T. molitor* larvae (Group B), a group supplemented with 0.05% chitosan (Group C), and a group supplemented with both 10% *T. molitor* larvae and 0.05% chitosan (Group D). The experimental period lasted 42 days (from 34 to 76 days of life). The results showed that *T. molitor* larvae supplementation improved the overall performance of the pigs, modified the fecal microbiota as well as the red blood cell content, and enhanced specific quality parameters of the meat such as phenolic content, oxidative stability, fatty acid profile, and color. Although chitosan supplementation did not affect the overall performance, it affected the blood lymphocyte count and the gut microbiota, while it also improved some meat quality parameters. Moreover, the combined supplementation of *T. molitor* and chitosan had positive effects on the zootechnical parameters, the fecal microbial populations, and the color and fatty acid profile of the meat.

**Abstract:**

Nowadays, the global animal industry faces considerable challenges in securing sufficient feed resources. Responding to consumer demands for reduced use of antibiotics in animal nutrition, better animal welfare status, and reduced impact on the environment, there is an increased urgency to develop innovative functional feeds with a reduced environmental footprint and the ability to improve meat quality and safety. In an effort to explore innovative feed ingredients for growing pig diets, the combined dietary supplementation of *Tenebrio molitor* larvae and chitosan was investigated. An experimental trial was performed with 48 weaned pigs (34 days of life; mixed sex) that were randomly assigned to four treatment groups (with six males and six females each): Group A (control), Group B (supplemented with *T. molitor* larvae at 10%), Group C (supplemented with chitosan at 0.05%), and Group D (supplemented with both ingredients at 10% and 0.05%, respectively). On the 42nd day of the experimental trial, samples of blood, feces, and carcass parts were taken for analysis. The results indicated that the insect larvae meal significantly improved (*p* < 0.05) overall performance, increased (*p* < 0.05) blood red blood cell content, increased meat phenolic content (*p* < 0.05), improved meat oxidative stability (*p* < 0.05), and affected meat fatty acid profile (*p* < 0.05). On the other hand, chitosan had no significant effect on overall performance (*p* > 0.05), but it significantly increased blood lymphocyte content (*p* < 0.05), affected the fecal microbiota (*p* < 0.05), improved meat oxidative stability (*p* < 0.05), increased meat phenolic content (*p* < 0.05), and affected meat fatty acid composition (*p* < 0.05) and (*p* < 0.05) meat color. Finally, the combined use of both *T. molitor* and chitosan significantly affected some important zootechnical parameters (*p* < 0.05), fecal microbial populations (*p* < 0.05), meat color (*p* < 0.05), and fatty acid profile (*p* < 0.05). Further investigation into the potential interaction between insect larvae meals and chitosan in pig diets is advised.

## 1. Introduction

The world’s human population is projected to surpass 9.6 billion by the year 2050, necessitating a 56% rise in global food demand. This rise is expected to result in heightened levels of hunger, as predicted by the United Nations [1] and Dijk et al. [2]. To tackle the imminent food crisis driven by this population growth, finding sufficient animal-based meat for consumers is an urgent matter. Consequently, future livestock production should focus on innovative methods when choosing potential feed sources. Ideally, these sources should be abundant, economically viable, and capable of providing nutrients comparable to traditional feed components.

Fishmeal and soybean meal are mainstay conventional protein components in diets for poultry and swine due to their well-balanced and highly digestible content of amino acids, fatty acids, vitamins, and minerals [3]. However, the constantly increasing production cost of these meals has raised the need for alternative high-protein feedstuffs such as insect meals [4].

Insects possess considerable potential for large-scale industrial production because of their ability for mass rearing, short life cycles, and efficient utilization of organic byproducts [5]. Organic waste, including expired feeds and food wastes, can serve as the feed for rearing mealworms, such as the yellow mealworm, *Tenebrio molitor* L. (Coleoptera: Tenebrionidae). Additionally, the protein and trace mineral content in mealworms is comparable to that of fishmeals and soybean meals [6].

Previously published research studies have indicated that incorporating *T. molitor* meal into pig diets has resulted in positive impacts on growth performance, gut microbiota, immune response, digestibility, and diarrhea rate [5,7,8,9]. This evidence strongly suggests that mealworms can be considered a viable source of high-quality protein in pig diets. However, further research is needed to clearly understand the effects of mealworm incorporation in livestock diets and to establish them as an alternative protein source.

Moreover, another major problem in the pork industry today is post-weaning stress. The period of weaning exposes piglets to sudden shifts in their environment, behavior, and diet [10]. This transition can lead to various negative outcomes, including gastrointestinal issues [11], dysfunctions in the immune system [12], and imbalances in the endocrine system [13]. These effects can be observed as diarrhea, increased vulnerability to illnesses, increased morbidity, and mortality [14,15], as well as inhibited growth and lower overall health.

Nowadays, post-weaning stress limitation is one of the main challenges for the pig industry. This is a multifactorial problem involving management, hygiene, and feeding parameters. The use of beneficial feed additives is one potential practice to mitigate post-weaning stress [10]. Recently, the use of chitosan in animal diets has been under examination due to its potential benefits to the health and growth performance of monogastric farm animals [16]. Chitosan is industrially manufactured through the process of deacetylating chitin, a structural component found in the exoskeletons of insects, crabs, and shrimps, as well as in the cell walls of fungi [17,18]. Specifically, chitosan derived from mushrooms presents greater advantages than that from crustaceans. This is because crustacean chitosan raises allergy concerns and is more expensive to produce [18]. Chitosan, which is a polymer composed of N-acetyl-D-glucosamine units, resembling plant cellulose in structure, is the second most abundant carbohydrate found in the natural world. [19]. These units are considered to have prebiotic properties, as they provide essential nutrients that assist the growth of useful gut bacteria [20], which play a pivotal role in the well-being of the digestive and immune systems of monogastric farm animals, ensuring the integrity of the intestinal mucosa and maximizing metabolic energy efficiency [21,22,23,24]. The published data suggest that *Streptococcus mutans* can metabolize N-acetyl-D-glucosamine [25].

Based on the available literature so far, the chitin content in *T. molitor* larvae ranges from 4.92% to 13.0%, with an average of 6.41% [26,27,28,29,30,31]. Due to this important chitin content mealworm larvae have the potential to serve as food or feed ingredients with prebiotic properties [9,32] that could modify the gastrointestinal microflora balance and offer health benefits to humans or animals. Moreover, the nutritional profile of *T. molitor*, with its essential proteins and amino acids, plays a crucial role in supporting animal growth and health [33]. When combined with the gut-enhancing properties of chitosan (part of the dried larvae meal or in pure form), these nutrients can be more effectively incorporated, giving synergistic effects, such as better growth performance and overall well-being of the animal.

According to the available published literature, there are limited data on the nutritional comparison of natural chitin of insect larvae versus high-purity chitosan. Also, the combined use of insect meals and high-purity chitosan has never been examined previously in swine diets. For these reasons, the aims of the present study were to compare a rich source of chitin, i.e., *T. molitor* larvae meal, to chitosan in the diets of young pigs and to evaluate whether their combined supplementation could indicate any antagonistic or synergistic effects on growth performance, gut microbiota, health status, and some quality parameters of the meat.

## 2. Materials and Methods

### 2.1. Experimental Design, Animals, and Diets

The Ethics and Research Ethics Committee of the University of Ioannina of Greece reviewed and approved the experimental protocol of this trial (protocol number 56652, 26 November 2021).

The experimental trial was performed on a commercial swine farm in the area of Epirus (Greece). Initially, 132 crossbred growing pigs (¼ Large White, ¼ Landrace, and ½ Duroc; 34 days of life) were examined to be clinically healthy by a veterinarian. From this initial pool, a total of 48 growing pigs (24 males and 24 females) with an average initial body weight of 8.38 ± 0.79 kg were randomly chosen and allocated into four distinct groups (groups A, B, C, and D; six males and six females per group) and housed in separated indoor pens with slatted plastic floors, heating, and mechanical ventilation. Each pig was uniquely identified with ear tags. Throughout the experiment, environmental factors including ambient temperature and humidity were constantly monitored (24–26 °C and 60–70%, respectively). Moreover, throughout the trial, the pigs had free access to fresh water and the feeds.

Larvae of *T. molitor*, reared in a conventional substrate (wheat bran), were utilized in the diets as a protein source. The larvae were raised for 120 days, beginning from newly hatched larvae until the stage of late-instar larvae, just before pupation, as described by Rumbos et al. [34]. The grown larvae were frozen and kept at −20 °C until us in the feed preparation. For chitosan supplementation, dry high-purity chitosan with a molecular weight of 250,000 daltons (GP8523-1000, Glentham Life Sciences Ltd., Corsham, UK) was procured.

Group A (the control group) was provided a typical feed based on maize, barley, and soybean meal, which was formulated following the guidelines of the NRC (National Research Council) [3] and the Premier Nutrition atlas [35]. For Group B, the *T. molitor* larvae meal was incorporated into the diet at a concentration of 100 g/kg as a replacement for the fishmeal. For Group C, the chitosan powder was added to the diet at a concentration of 0.5 g/kg. In the diet of Group D, both the *T. molitor* larvae meal (100 g/kg) and chitosan (0.5 g/kg) were added to the diet. The four diets were formulated to be isocaloric and isonitrogenous. On the day that the diets were prepared, the *T. molitor* whole larvae were taken out of the freezer, weighed, ground in a hammer mill together with the other feed ingredients, and mixed in a horizontal mixer according to the common procedure performed in the commercial farm. Based on the available literature, dried *T. molitor* larvae contain on average 11.56 g/kg of chitosan [9], and using this value, the chitosan content of the diets for Groups B and D was calculated. Table 1 presents the chemical composition of *T. molitor* whole larvae meal. Table 2 presents the ingredients and chemical composition of the four diets.

The feeding trial lasted 42 days. Throughout this period, the growing pigs were weighed individually on the 1st, 21st, and 42nd days using a Mini-L 3510 animal scale (Zigisis, Chalkidiki, Greece). Daily records were maintained for feed intake and any cases of disease or mortality. The performance parameters that were evaluated during the trial included average weight, average weight gain, average feed intake, and average feed conversion ratio (FCR, calculated as kg of feed intake per kg of live weight gain) for three specific periods: 1–21 days, 21–42 days, and the overall period of 1–42 days. On the 42nd day, six animals were randomly selected from each pen, were processed in a nearby commercial abattoir, and their carcasses were stored.

### 2.2. Determination of Fecal Microbial Populations

On the 42nd day of the experiment, fresh fecal (stool) samples were collected from each growing pig for microbial analysis as described by Choudhury et al. [36]. The mediums used for the determination of the different bacterial populations were plate count agar medium (Oxoid, Basingstoke, UK) for the total aerobic and anaerobic bacterial counts; MacConkey (McC) and kanamycin aesculin azide (KAA) agar (Merck, Darmstadt, Germany) for the Enterobacteriaceae and Enterococcaceae; and De Man, Rogosa, and Sharpe (MRS) agar (Oxoid, Basingstoke, UK) and M17 agar (Lab M Limited, Lancashire, UK) for the Lactobacillaceae.

Bacterial enumerations were performed using the surface drop technique and the results were expressed as Log_10_ of colony-forming units (CFU) per 1 g of wet-weight sample. To identify the bacterial populations at the family level, an automated “Vitek 2 compact system” (bioMérieux, Marcy l’Etoile, France) was utilized [37].

### 2.3. Hematological and Biochemical Analysis of Blood Samples

On the 42nd day of the experiment, the feeders of the pigs were emptied four hours before blood sampling. Samples (4 mL) of blood were collected from the jugular veins of six animals per group and deposited into vacutainer tubes containing ethylenediaminetetraacetic acid as an anticoagulant agent. The hematological parameters (white blood cells, lymphocytes, monocytes, granulocytes, red blood cells, hematocrit, hemoglobin, and thrombomodulin), were analyzed with an “MS4 automated analyzer” (Melet Schloesing Lab, Osny, France) [38]. Additionally, some serum biochemical parameters (albumin, alanine aminotransferase, aspartate aminotransferase, cholesterol, creatine kinase, glucose, total bilirubin, and triglycerides) were determined with an “IDEXX VETTEST 8008 analyzer” (IDEXX LAB, Westbrook, ME, USA) [39].

### 2.4. Collection of Meat Samples

The pigs were transported to the nearby commercial abattoir and then were processed following the regulations set forth by the national guidelines [40]. During the processing, the carcasses were weighed and then meat samples were collected from four specific muscle areas: ham (*biceps femoris* and *semimembranosus* muscles), shoulder (*trapezius* and *triceps branchi* muscles), belly (*external abdominal* and *oblique* muscles), and boneless steak (*longissimus thoracis* muscle).

### 2.5. Microbial Analysis of Meat Samples

The microbial populations of the samples of the shoulder, belly, and boneless steak cut were identified and counted using conventional methods, following the procedure described by Bonos et al. [41]. In addition, the presence of *Salmonella* spp. and *Listeria monocytogenes* (per 25 g of sample) was tested according to the procedures of ISO 6579:2002 and ΙSO 4833:2001, respectively [42,43].

### 2.6. Chemical Analysis of Meat Samples

All collected meat cuts were stored at −20 °C to preserve their freshness and prevent any spoilage. On the day of the analysis, subsamples of 200 g were cut, unfrozen, and minced using a commercial meat mincer (Bosch, Gerlingen, Germany). Moisture, crude protein, fat, collagen, and ash content were analyzed with a “FoodScanTM Lab analyzer” (FOSS, Hillerod, Denmark), following the AOAC 2007.04 guidelines [44,45].

### 2.7. Total Phenolic Analysis of Meat Samples

A modified Folin–Ciocalteu method [46] was utilized to measure the total polyphenols in the meat subsamples from the shoulder, belly, and boneless steak, following the procedure described by Vasilopoulos et al. [47].

### 2.8. Oxidative Stability Analysis of Meat Samples

Lipid oxidation in meat samples was evaluated by the thiobarbituric acid reactive substances (TBARS) method, based on Ahn et al. [48] but with slight alterations. Briefly, 5 g of shoulder and belly meat samples from carcasses refrigerated at 4 °C for 1 and 7 days were taken and blended in an Ultra-Turrax T25 device (Janke & Kunkel, IKA Labortechnik, Staufen, Germany) with 15 mL of distilled water. From this, 5 mL portions were combined with 50 mL of 7.2% butylated hydroxyanisol in test tubes. Subsequently, 5 mL of TBA (a solution combining 20 mM trichloroacetic acid with 15% trichloroacetic acid) was added to the tubes. These mixtures were then vigorously stirred, heated in a water bath at 100 °C for 15 min, cooled, and centrifuged at 1000× *g* at 4 °C for 15 min. The absorbance of each resultant organic layer was measured using a spectrophotometer UV 1700 (PharmaSpec, Shimadzu, Japan) at 532 nm. The extent of lipid oxidation was quantified using the TBARS value, expressed in terms of nanograms of malondialdehyde per gram of meat.

### 2.9. Color and pH Analysis of Meat Samples

The chromatic profiles of the meat cuts (shoulder, belly, and boneless steak) were assessed using the “Hunter scale” (L*, a*, and b* values) using a “CAM-System 500” analyzer (Lovibond, Amesbury, UK) and following the method described by Bonos et al. [41].

The pH levels of these meat cuts were assessed using a “Hanna Instruments pH meter” (Woonsocket, RI, USA), as described by Van de Perre et al. [49].

### 2.10. Meat Fatty Acid Analysis

To analyze the fatty acid profile of the shoulder meat samples, the processing method described by O’Fallon et al. [50] was followed. The separation and quantification of the methyl esters were carried out by following the procedure of Skoufos et al. [51]. The analysis was conducted using a “TraceGC device Model K07332” (Thermofinigan, Thermoquest, Milan, Italy), which was equipped with a flame ionization detector (FID).

### 2.11. Statistical Analysis

The study was designed using a random complete block design (RCB), where the experimental unit was the individual growing pig. The experimental data were evaluated using a two-way analysis of variance (ANOVA) that tested the effects of insect larvae meal supplementation (without supplementation vs. with supplementation) and chitosan supplementation (without supplementation vs. with supplementation) as fixed factors, as well as their interaction. The analysis was performed using the general linear model function of the SPSS v20 statistical package (IBM, Armonk, NY, USA) [52]. To prepare the microbiology data for analysis, a log transformation (log_10_) was applied. Data homogeneity was checked using Levene’s test and data normality using the Shapiro–Wilk test. The significance level was set at 5% (*p* ≤ 0.05). In cases where a significant interaction and significant differences between the fixed factors were observed, Tukey’s post hoc test (*p* ≤ 0.05) was used for pairwise comparisons among the four experimental groups.

## 3. Results

### 3.1. Performance Parameters

Table 3 presents the effects of the different diets on the performance parameters. *T. molitor* supplementation resulted in significantly increased body weight at day 21 (*p* < 0.001) and at day 42 (*p* = 0.011) and higher weight gain for the periods from 1 to 21 days (*p* < 0.001) and 1 to 42 days (*p* = 0.015). Moreover, chitosan supplementation resulted in higher carcass weight of the growing pigs (*p* = 0.041). Significant interactions between *T. molitor* content and chitosan supplementation were found for body weight at day 42 (*p* = 0.017) and weight gain for the periods from 21 to 42 days (*p* = 0.014) and 1 to 42 days (*p* = 0.017). Based on the interaction analysis, it should be noted that the combined use of the two additives significantly increased (*p* ≤ 0.05) the body weight at day 42 (Group D vs. Group C) and weight gain for the period from 1 to 42 days (Group D vs. Groups A and C). In addition, the exclusive use of chitosan significantly (*p* < 0.05) decreased weight gain for the period from 21 to 42 days. No significant differences (*p* > 0.05) were observed for the other performance parameters. Furthermore, there were no mortalities and no identified cases of clinical disease throughout the experimental period.

### 3.2. Fecal Microbial Populations

The effects of dietary supplementation on fecal microbial species are shown in Table 4. The fecal microflora was not affected by *T. molitor* supplementation (*p* > 0.05). Chitosan supplementation resulted in lower Enterobacteriaceae counts (*p* = 0.014), lower total aerobic bacteria (*p* < 0.001), and higher total anaerobic bacteria (*p* = 0.031). Significant interactions between *T. molitor* and chitosan supplementation were found for Lactobacillaceae counts (*p* = 0.010) and total aerobic bacteria (*p* = 0.002). Based on the interaction analysis, it should be noted that the combined use of the two additives significantly (*p* < 0.05) increased Lactobacillaceae counts compared to the exclusive supplementation of *T. molitor* (Group D vs. Group B). Furthermore, total aerobic bacteria were significantly (*p* < 0.05) decreased by the combined supplementation (Group D vs. Groups A, B, and C) and, to a lesser degree, by the stand-alone chitosan supplementation (Group C vs. Groups A, B). Enterococcaceae were not affected either by the supplementation of chitosan or by the combined supplementation.

### 3.3. Blood Parameters

Table 5 shows the effects of dietary supplementation on the examined hematological and biochemical parameters. Red blood cells were significantly (*p* = 0.050) increased by *T. molitor* supplementation, whereas chitosan supplementation significantly increased lymphocyte counts (*p* = 0.029) and blood triglycerides (*p* = 0.030). Significant interactions between *T. molitor* and chitosan supplementation were observed only on monocyte counts (*p* = 0.029). Exclusive supplementation of only *T. molitor* or chitosan significantly (*p* ≤ 0.05) decreased monocyte counts (Groups B and C vs. Group A), whereas this effect was not apparent in the combined use of these two additives. No significant differences (*p* > 0.05) were observed for the other examined parameters.

### 3.4. Microbiological, Chemical, and Oxidative Stability Analysis of the Meat Samples

Table 6 presents the microbial analysis of the meat samples. The feed with *T. molitor* supplementation resulted in decreased *E. coli* (*p* < 0.001) and *C. jejuni* (*p* = 0.004) populations of the shoulder meat cut, whereas no differences were noted for the other meat cuts. The use of chitosan lowered (*p* = 0.019) the *Staphylococcus* spp. populations in the shoulder meat cut but did not affect (*p* > 0.05) the microbial populations of the other meat cuts. No significant interaction was found between the two feed ingredients regarding the microbial populations of the examined meat cuts.

Furthermore, Table 7 shows the chemical composition analysis of the meat samples. *T. molitor* supplementation significantly (*p* = 0.009) decreased the moisture content of the ham meat cut and the ash content (*p* = 0.015) of the belly meat cut. Chitosan supplementation resulted in decreased (*p* = 0.041) ash content of the boneless steak meat cut. No significant interactions were found between the two ingredients for the chemical composition of the examined meat cuts.

The effects of the dietary supplementation with *T. molitor* larvae meal and chitosan on the total phenolic content, oxidative stability, pH, and color of the meat samples are given in Table 8. *T. molitor* supplementation significantly increased (*p* = 0.010) the total phenolic content of shoulder meat, decreased (*p* = 0.042) the MDA content in belly meat on Day 1, and decreased (*p* = 0.001) the MDA content in shoulder meat on Day 7. Chitosan supplementation significantly increased (*p* = 0.013) the total phenolic content of belly meat, decreased (*p* = 0.002) the MDA content in belly meat on Day 1, decreased (*p* = 0.002) the MDA content in shoulder meat on Day 7, decreased (*p* = 0.002) the pH of belly meat, increased (*p* = 0.032) the pH of boneless steak meat, decreased (*p* = 0.036) the L* (lightness) value of shoulder meat, and increased (*p* = 0.034) the b* (yellowness) value of boneless steak meat. Based on the interaction analysis, it should be noted that both the exclusive and the combined use of the two additives significantly decreased (*p* < 0.05) the MDA content in shoulder meat on Day 7 (Groups B, C, and D vs. Group A). However, only the exclusive use of *T. molitor* meal decreased (*p* < 0.05) the MDA content in belly meat on Day 7 (Group B vs. Group A). Finally, the exclusive use of chitosan increased (*p* ≤ 0.05) the b* (yellowness) value of belly meat (Group C vs. Group A).

The effects of the dietary supplementation on the shoulder meat fatty acid profile are presented in Table 9. *T. molitor* supplementation significantly increased C17:1 (cis-10-heptadecenoic; *p* = 0.029), C18:2n6c (linoleic; *p* = 0.001), C21:0 (heneicosanoic; *p* = 0.009), and total polyunsaturated (*p* = 0.001), and omega-6 (*p* = 0.001) fatty acids in the meat, whereas this supplementation decreased C14:0 (myristic; *p* = 0.002), C15:0 (pentadecanoic; *p* = 0.028), C18:0 (stearic; *p* = 0.039), and total saturated (*p* = 0.006) fatty acids. Chitosan supplementation significantly increased C16:1 (palmitoleic; *p* < 0.001), C18:1n9t (elaidic; *p* = 0.001), C18:1n9c (oleic; *p* = 0.003), C18:3n6 (γ-linolenic; *p* = 0.036), C20:0 (arachidic; *p* = 0.002), C18:3n3 (a-linolenic; *p* = 0.049), monounsaturated (*p* = 0.001), and omega-3 (*p* = 0.049) fatty acids in the meat, whereas this supplementation decreased C14:0 (myristic; *p* = 0.004), C15:1 (cis-10-pentadecenoic; *p* = 0.007), C17:1 (cis-10-heptadecenoic; *p* = 0.002), C22:0 (behenic; *p* = 0.001), and total saturated (*p* = 0.002) fatty acids. Significant interactions between *T. molitor* and chitosan supplementation were identified for the C14:0 (myristic; *p* = 0.004), C15:0 (pentadecanoic; *p* = 0.048), and C16:1 (palmitoleic; *p* = 0.001) fatty acids. Based on the interaction analysis, the C14:0 (myristic) fatty acid was lower in the meat of all supplemented treatments (Groups B, C, and D vs. Group A). Although the C15:0 (pentadecanoic) fatty acid was decreased by the addition of *T. molitor* (Groups B and D vs. Group A), this reduction seemed to be alleviated by chitosan supplementation (Group C). C16:1 (palmitoleic) fatty acid was increased by both the exclusive and combined use of the two additives (Groups B, C, and D vs. Group A).

## 4. Discussion

Over the past several decades, there has been a significant enhancement in the growth rate of growing pigs. This has led to achieving greater body weight in a shorter period of time and an improved feed conversion ratio. However, the practice of early weaning—around 3–4 weeks—presents both nutritional and environmental challenges and can often negatively affect the gut functionality, immunity, feed efficiency, and overall growth performance of the piglets [53]. In response, the industry has tried to improve the formulations of the diets for piglets, trying to achieve high palatability and digestibility by utilizing highly digestible animal protein sources, such as insect meals, and promote good growth and health. These diets are expected to fulfill the dietary needs and promote the growth of the gastrointestinal and immune systems in young pigs [54]. To our knowledge, the present study is the first that investigates the combined use of *T. molitor* larvae meal and chitosan in growing pig diets. The results of the trial suggest that employing a combination of *T. molitor* larvae meal (100 g/kg) and chitosan (0.5 g/kg) can completely replace 30 g/kg fishmeal in the diets of early-weaned growing pigs, simultaneously improving their performance parameters. Some previously published studies have already demonstrated improvements in zootechnical parameters when *T. molitor* was incorporated into growing pig diets [5,9]. However, it is important to highlight that not all studies have reported consistent results. For instance, Meyer et al. indicated a negative impact on pig growth performance when *T. molitor* was included in the final diet at a 10% rate [55]. From this perspective, chitosan appears pivotal to the pigs’ enhanced performance. This improvement in zootechnical parameters is likely due to the additional chitosan supplementation combined with the chitin contained in the *T. molitor* larvae; notably, chitosan, due to its molecular structure, resists mammalian enzyme digestion and reaches the large intestine mostly unaltered. In the present experiment, while Group A had no chitosan content, the other groups had chitosan added either as part of the dried larvae meal or in pure form. More specifically, this combined addition increased the amount of chitosan in Group D by 43% compared to Group B. Chitosan is considered to possess prebiotic properties and is known to enhance gut health and consequently animal performance. Diets enriched with chitosan and tested in the growing pigs resulted in an enhanced growth rate, while this effect was attributed to the elevated levels of digestive enzymes (such as amylase) and the improved health status of the epithelium of the small intestine [10,56]. In addition, these results suggest a potential boost in nutrient digestibility. Another important consideration is that insects not only recycle waste into protein but also have a lower carbon footprint and an efficient feed conversion ratio [57]. However, in the EU, the utilization of catering or animal waste as insect feed is prohibited [58]. Based on these results, using *T. molitor* larvae offers a promising alternative protein source for pig farming with improved sustainability processes.

During the weaning transition, piglets often face both various external environmental pressures and internal physiological challenges [59]. Weaned piglets, in particular, are vulnerable to pathogens because their intestinal immune system is not fully developed, which is often identified as an imbalance in the growth of intestinal beneficial versus potentially pathogenic microorganisms [60]. Currently, considerable research efforts are focused on seeking effective feed additives that can regulate the gut microbiota and alleviate intestinal inflammation. The gut microbiota is a complex and critical system that plays a vital role in numerous physiological functions of the host, including intestinal structure, barrier function, immune response, and overall health [61,62]. A primary factor influencing intestinal microbiota is the nutrient composition of the diet, particularly the incorporation of antimicrobial compounds, whether they are of natural or synthetic origin [63]. In this study, we investigated the bacterial composition of the pig feces using a culture-dependent techniques analysis. Incorporating chitosan as a dietary supplement in the feeding trial resulted in a reduction in Enterobacteriaceae and total aerobic bacteria counts, while concurrently increasing the count of total anaerobic bacteria, which is consistent with previous reports [24,64]. Based on these results, it can be extrapolated that the supplementation of chitosan has a positive impact on gut microbial populations. This is supported by the decrease in total aerobic counts, which typically include various potentially pathogenic microorganisms [65]; from the increase in total anaerobic bacteria, which are capable of producing short-chain fatty acids [66]; and from the reduction in Enterobacteriaceae, which include some of the most pathogenic bacteria in pig production [67], leading to substantial economic losses. These modifications may be associated with chitosan, which can act as a distinct nutrient for certain families of intestinal microbes. Consequently, chitosan supplementation can cause changes in the types of microbial fermentation metabolites produced within the intestinal lumen [68]. Moreover, insect meals are rich in antimicrobial compounds that can inhibit the growth of pathogenic bacteria, such as methicillin-resistant *Staphylococcus aureus* [69]. Additionally, Choi et al. report that the use of insect-derived antimicrobial peptides, such as AMP-P5, in monogastric animal diets yields results on the intestinal microbiota comparable to those of antibiotics [70]. Furthermore, in the present experiment, the combined use of *T. molitor* and chitosan increased Lactobacillaceae counts and decreased total aerobic bacteria. Lactobacillaceae is a family of bacteria that can have several beneficial effects on gut health, immune support, and nutrient absorption [71]. The stress of weaning pigs often leads to changes in their gut bacteria: a decrease in beneficial bacteria like the *Lactobacillus* group and a reduction in microbial diversity create favorable conditions for the growth of harmful bacteria like *Clostridium* spp., *Prevotella* spp., and *Escherichia coli* [72]. Notably, in our experiment, the combined supplementation with *T. molitor* larvae meal and chitosan had a beneficial effect on the examined fecal microbiota. This suggests that this combination can potentially alleviate post-weaning stress in growing pigs.

Hematological traits serve as crucial indicators for evaluating an animal’s overall health and physiological condition, providing insights into its physiological responsiveness [73]. In the current investigation, a comprehensive analysis of hematological and biochemical parameters showed that all the evaluated parameters remained within the established physiological ranges for swine, serving as a robust indicator of the animals’ overall health. This suggests that the test diets were of sufficient quality to sustain the well-being of the pigs, thereby supporting the potential viability of the alternative ingredients under study. The use of *T. molitor* increased the red blood cell counts, which is clear evidence of more efficient erythropoiesis in the experimental pigs [74]. Our results are in accordance with those of Chia et al. [75], who observed that incorporating insect meal into the diets of growing pigs led to elevated red blood cell counts. This enhancement may be accredited to the good digestibility of insect-derived proteins, as well as the abundant mineral content, particularly iron, which is essential for hemoglobin synthesis in pigs. Furthermore, our experimental trial revealed that chitosan supplementation led to elevated lymphocyte levels. The condition of the immune system is determined by white blood cells like lymphocytes, which are critical contributors to the immune defenses of both humans and animals [76]. It is well established that chitosan plays a role in enhancing the immune functions of lymphocytes by promoting the upregulation of key cytokines such as interleukin-1, tumor necrosis factor-alpha, and interferon-gamma [77,78]. This indicates that chitosan has the ability to activate both cellular and humoral immune defenses in pigs.

The presence of microbes in meat is strictly connected to its overall quality and safety for consumption. Although muscle tissues are usually sterile in live animals, under commercial processing conditions, their meat is contaminated during slaughter, cutting, and storage. In the present study, all measured levels of microbial contamination fell within acceptable safety parameters. Additionally, we found no evidence of harmful bacteria such as *Salmonella* spp. or *Listeria monocytogenes* in any of the 25 g meat samples analyzed. The only statistically significant result was the reduction in *Escherichia coli* and *Campylobacter jejuni* especially in the shoulder meat samples when *T. molitor* whole larvae meal was used. The reported antimicrobial activity of insect meals is linked to their rich content of antimicrobial peptides [79]. Additionally, according to Chen et al. [80], there is a remarkable association between the intestinal microbial populations and the quality of swine meat, suggesting that the diet of an animal has the potential to influence not only the gut microbial communities but also the bacterial metabolites and, consequently, the overall quality of the meat during storage. Also, it has been documented that elevated populations of beneficial gut bacteria are positively associated with superior meat quality [81]. Finally, the present results are in agreement with previous studies, which reported that the dietary use of *T. molitor* larvae decreased the populations of potentially harmful bacteria such as *E. coli*, *Clostridium* spp., and *Staphylococcus* spp. in meat cuts of growing pigs [82].

Meat quality is vital to the economic viability of pig farming, as it directly influences the meat’s capacity for extended storage and further processing [83]. The impact of alternative production systems on the chemical composition of pig meat is not consistently supported by the existing literature. Generally, physical activity affects certain meat quality traits, such as muscle metabolism and post-slaughter pH levels, more than it does the meat’s chemical composition [84]. When there are changes in the meat’s chemical composition, they are often due to management factors like feed composition, feed intake, and the metabolic energy used for maintenance [85]. Recent studies have specifically explored how alternative production systems influence the chemical properties of meat [86]. In our study, the dietary use of *T. molitor* and chitosan did not affect the main parameters of the chemical composition of the meat cuts, such as fat, protein, and collagen. It can be noted that the use of *T. molitor* decreased the moisture content of the ham and the ash content of the belly. In addition, the ash content of boneless steak was decreased by the use of chitosan. The increase in moisture content in meat may be attributable to its inverse relationship with fat content, as originally outlined by Callow [87]. These two factors are closely connected to the meat’s juiciness. In pigs, a higher percentage of lean meat is associated with a higher level of ash content and a lower level of intramuscular fat [88]. The present results diverge from previous research [89] that suggested the inclusion of *T. molitor* in pig feed leads to higher percentages of protein and fat in pork meat. In conclusion, the present research found that using *T. molitor* and chitosan in pig feed does not have a negative impact on meat quality. It should be noted that these ingredients offer promising opportunities for further study, particularly in exploring how they affect the deposit of minerals in muscle tissue.

It is well established that the animal diet plays a significant role in shaping the physicochemical properties of the produced meat [90,91]. Conducting tests to measure the antioxidant capacity is particularly valuable for assessing the antioxidant status of meat from animals that have been given different types of feed [92]. In the present experiment, the total phenolic value was increased by the supplementation of *T. molitor* and chitosan in most meat cuts. Respectively, MDA content was reduced at Days 1 and 7 under refrigeration in most meat cuts. It appears that there is a relationship between the content of dietary phenols in the meat and its resistance to oxidative damage. These observations are in agreement with previous studies, which reported that the use of *T. molitor* in monogastric animal diets can improve the oxidative stability of the meat [47,82]. Xu et al. reported that incorporating chitosan into the diets of weaned piglets led to an increase in total antioxidant capacity along with a reduction in the levels of MDA and cortisol in serum [10]. However, supplementing *T. molitor* into the feeds of growing pigs did not influence the thiobarbituric acid reactive substances in ham cuts [7]. These results indicate the potential of both *T. molitor* and chitosan to protect growing pigs from oxidative stress by enhancing the functions of their antioxidant defense systems.

The quality of swine meat is intricately connected to the pH levels in the edible tissue [93]. Additionally, consumer decisions to purchase meat are frequently influenced by the visual appeal of its color [94]. The incorporation of chitosan led to contrasting effects on pH values in different meat cuts: it lowered the pH in belly meat while raising it in boneless steak cuts. However, it is important to note that the pH levels for all examined meat cuts fell within the preferred acceptable ranges [95]. In addition, the present results show significant alterations in L* and b* color values of some meat cuts, particularly with chitosan supplementation. Enhanced lightness, represented by the L* value, may be associated with the oxidation of myoglobin to metmyoglobin [96]. Therefore, the decrease in MDA content in shoulder meat due to the application of chitosan could be correlated with the reduction in lightness for this meat cut. Some authors have reported that adding dietary chitin and its byproducts, such as chitosan and chito-oligosaccharides, could positively affect certain parameters of pig meat quality, for example, drip loss and color [97]. However, the supplementation of chitosan in the present study led to an increase in the b* value for boneless steak cuts, suggesting that the color quality of the meat is affected by chitosan supplementation. It is important to remember that an elevated b* value in pork meat is generally considered disadvantageous, as it indicates abnormal meat color and has been positively linked to pale, soft, exudative (PSE) pork meat [98,99].

The fatty acid profile of meat from monogastric animals, such as pigs, is directly shaped by the specific types of fats included in their diet [100]. In the current study, the fatty acid composition of the meat was altered by the dietary supplementation of both *T. molitor* and chitosan. Supplementation with *T. molitor* resulted in reduced levels of total saturated fatty acids and elevated levels of total polyunsaturated and omega-6 fatty acids in shoulder meat cuts. According to Siemianowska et al. [101], the fatty acid profile of *T. molitor* larvae is notably rich in monounsaturated fatty acids, specifically oleic, elaidic, linoleic, and eicosapentaenoic acids. Moreover, the fatty acid composition of *T. molitor* larvae and meals can be improved through modification of the rearing substrate [102]. Our results are in agreement with recent studies, which also reported a decrease in saturated fatty acids (SFA) and an increase in polyunsaturated and omega-3 fatty acids in the produced meat of growing pigs when *H. illucens*, *T. molitor*, and *A. diaperinus* larvae meals were added in the diets [82,103,104,105]. In contrast, there is limited research on the effect of chitosan supplementation on pork meat fatty acids. Chitosan supplementation decreased total saturated and increased total monounsaturated and omega-3 fatty acids in the shoulder meat cut. A reasonable explanation could be that chitosan not only boosts the synthesis of short-chain fatty acids [106] but also improves lipid metabolism [107].

## 5. Conclusions

This study evaluated the synergistic impact of *T. molitor* whole larvae meal and chitosan supplementation on the diets of growing pigs. Our results on the combined use of these ingredients are particularly encouraging, demonstrating not only enhanced growth parameters but also beneficial effects on gut microbial populations, hematological parameters, and some identified effects in meat quality parameters (such as fatty acid composition). While further investigation is needed to fully validate the efficacy of the combined use of insect meals and chitosan in swine nutrition, our study provides strong evidence for the benefits of this innovative dietary approach.

## Figures and Tables

**Table 1:** Chemical composition of *T. molitor* whole larvae meal.

Chemical Composition, g/kg as Fed	*T. molitor* Whole Larvae Meal
Dry matter	271.6
Digestible energy (DE, MJ/kg)	7.6
Crude protein	169.8
Crude fiber	22.0
Ether extract	123.0
Ash	13.0
Acid detergent fiber (ADF)	23.0
Neutral detergent fiber (NDF)	52.0
Chitin	11.56
Lysine	10.0
Meth + Cyst	5.0
Methionine	0.3
Cystine	0.2
Threonine	10.0
Tryptophan	1.9
Calcium	1.0
Total phosphorus	3.0

**Table 2:** Ingredients and chemical composition of the four experimental feeds.

Ingredients, g/kg as Fed	Group A	Group B	Group C	Group D
Maize	336.0	205.5	335.5	205.0
Barley	347.0	347.0	347.0	347.0
Wheat middlings	30.0	30.0	30.0	30.0
Soybean meal (47% crude protein)	168.0	188.8	168.0	188.8
Soybean oil	19.0	54.8	19.0	54.8
Vitamin and mineral premix ^1^	60.0	60.0	60.0	60.0
Fishmeal (72% crude protein)	30.0	0.0	30.0	0.0
*T. molitor* whole larvae meal	0.0	100.0	0.0	100.0
Chitosan	0.0	0.0	0.5	0.5
Benzoic acid	3.0	3.0	3.0	3.0
Zn oxide	3.0	3.0	3.0	3.0
Salt	2.0	2.0	2.0	2.0
Monocalcium phosphate (22% P)	2.0	6.0	2.0	6.0
**Calculated analysis, g/kg as fed**				
Dry matter	884.2	841.6	884.2	841.6
Digestible energy (DE, MJ/kg)	13.6	13.6	13.6	13.6
Crude protein	186.6	186.5	186.6	186.5
Crude fiber	34.5	34.9	34.5	34.9
Ether extract	39.4	79.0	39.4	79.0
Ash	52.8	54.1	52.8	54.1
Acid detergent fiber (ADF)	39.5	39.8	39.5	39.8
Neutral detergent fiber (NDF)	114.0	109.0	114.0	109.0
Chitosan	0.000	1.156	0.500	1.656
Lysine	12.3	12.2	12.3	12.2
Meth + Cyst	7.7	7.4	7.7	7.4
Methionine	4.9	4.6	4.9	4.6
Cystine	2.8	2.8	2.8	2.8
Threonine	6.2	6.5	6.2	6.5
Tryptophan	2.0	2.1	2.0	2.1
Calcium	5.6	5.5	5.6	5.5
Total phosphorus	5.0	5.3	5.0	5.3
Sodium	3.0	2.9	3.0	2.9
Chloride	5.2	4.9	5.2	4.9
Potassium	6.7	6.4	6.7	6.4

^1^ Supplied per kg diet: 15,000 IU retinol, 50 mcg 25-hydroxyvitamin D3, 9.96 mg tocopherol, 10.02 mg menadione, 3 mg thiamine, 10.02 mg riboflavin, 6 mg pantothenic acid, 6 mg pyridoxine, 40.02 mcg cobalamin, 100 mg ascorbic acid, 35 mg nicotinic acid, 300 mcg biotin, 1.5 mg folic acid, 375 mg choline chloride, 200 mg iron (II) sulfate monohydrate, 90 mg copper sulfate pentahydrate, 60 mg manganese sulfate monohydrate, 100 mg zinc sulfate monohydrate, 2 mg calcium iodate, 300 mg sodium selenide, 150 mg L-selenomethionine–selenium, 1500 FYT 6-phytase, 80 U β-1,4-endoglucanase, 70 U β-1,3 (4)-endoglucanase, 270 U β-1,4-endoxylanase, 5000 mg benzoic acid, 40.8 mg butylated hydroxytoluene, 3.5 mg propyl gallate.

**Table 3:** Effect of dietary addition of *Tenebrio molitor* larvae meal and chitosan on performance parameters of growing pigs.

Body Weight on Day (kg)	Treatments					Effect of *T. molitor* Meal			Effect of Chitosan			Interaction
	Group A	Group B	Group C	Group D	SEM	Without *T. molitor*	With *T. molitor*	*p*	Without Chitosan	With Chitosan	*p*	*p*
1	8.41	8.51	8.31	8.31	0.114	8.36	8.41	0.831	8.46	8.31	0.525	0.833
21	14.77	16.86	15.15	17.46	0.277	14.96	17.16	<0.001	15.81	16.26	0.298	0.814
42	24.86 ^ab^	24.98 ^ab^	23.12 ^a^	26.96 ^b^	0.412	23.95	25.93	0.011	24.92	24.88	0.875	0.017
**Weight gain for period (kg)**												
1 to 21 days	6.36	8.35	6.84	9.15	0.231	6.61	8.75	<0.001	7.35	7.95	0.066	0.640
21 to 42 days	10.09 ^b^	8.13 ^ab^	7.97 ^a^	9.61 ^ab^	0.368	8.99	8.83	0.819	9.11	8.72	0.654	0.014
1 to 42 days	16.45 ^a^	16.48 ^ab^	14.81 ^a^	18.63 ^b^	0.417	15.60	17.50	0.015	16.46	16.56	0.739	0.017
**Feed intake per pig for period (kg)**												
1 to 21 days	14.56	14.02	13.53	13.87	-	-	-	-	-	-	-	-
21 to 42 days	21.19	20.46	19.65	20.25	-	-	-	-	-	-	-	-
1 to 42 days	35.75	34.48	33.18	34.12	-	-	-	-	-	-	-	-
**Feed conversion ratio (kg feed/kg weight gain)**												
1 to 21 days	2.29	1.68	1.98	1.52	-	-	-	-	-	-	-	-
21 to 42 days	2.10	2.52	2.47	2.30	-	-	-	-	-	-	-	-
1 to 42 days	2.17	2.09	2.24	1.82	-	-	-	-	-	-	-	-
**Carcass parameters**												
Carcass weight (kg)	14.94	15.66	16.72	18.26	0.538	16.72	15.83	0.272	15.30	17.49	0.041	0.685
Carcass dressing percentage (%)	0.63	0.63	0.74	0.67	0.022	0.68	0.65	0.420	0.63	0.70	0.103	0.394

Group A, control diet; Group B, diet with 100 g/kg *T. molitor* meal; Group C, diet with 0.5 g/kg chitosan; Group D, diet with 100 g/kg *T. molitor* meal and 0.5 g/kg chitosan. *N* = 12 per treatment. ^a,b^ Mean values with different superscripts are significantly (*p* ≤ 0.05) different.

**Table 4:** Effect of dietary addition of *Tenebrio molitor* larvae meal and chitosan on fecal microflora populations of growing pigs.

Day 42 (Log_10_ CFU/g)	Treatments					Effect of *T. molitor* Meal			Effect of Chitosan			Interaction
	Group A	Group B	Group C	Group D	SEM	Without *T. molitor*	With *T. molitor*	*p*	Without Chitosan	With Chitosan	*p*	*p*
Enterobacteriaceae	6.46	6.90	6.08	5.89	0.141	6.29	6.39	0.642	6.66	5.99	0.014	0.251
Enterococcaceae	4.06	4.06	3.87	3.88	0.098	3.99	3.95	0.982	4.06	3.87	0.413	0.997
Lactobacillaceae	8.12 ^ab^	6.96 ^a^	7.44 ^ab^	8.60 ^b^	0.228	7.81	7.78	0.988	7.59	8.02	0.275	0.010
Total aerobes	8.34 ^c^	8.63 ^c^	7.43 ^b^	6.64 ^a^	0.143	7.93	7.68	0.128	8.48	7.03	<0.001	0.002
Total anaerobes	8.56	8.74	8.93	9.23	0.100	8.73	8.97	0.226	8.65	9.08	0.031	0.744

Group A, control diet; Group B, diet with 100 g/kg *T. molitor* meal; Group C, diet with 0.5 g/kg chitosan; Group D, diet with 100 g/kg *T. molitor* meal and 0.5 g/kg chitosan. *N* = 12 per treatment. ^a, b, c^ Mean values with different superscripts are significantly (*p* ≤ 0.05) different.

**Table 5:** Effect of dietary addition of *Tenebrio molitor* larvae meal and chitosan on blood parameters of growing pigs.

Hematological Parameters	Treatments					Effect of *T. molitor* Meal			Effect of Chitosan			Interaction
	Group A	Group B	Group C	Group D	SEM	Without *T. molitor*	With *T. molitor*	*p*	Without Chitosan	With Chitosan	*p*	*p*
WBC (m/mm^3^)	23.47	22.03	23.44	21.00	1.052	23.45	21.51	0.390	22.75	22.22	0.813	0.823
Lym (%)	34.33	35.48	38.82	39.08	0.907	36.58	37.28	0.684	34.91	38.95	0.029	0.799
Mon (%)	9.35 ^b^	7.58 ^a^	7.63 ^a^	8.80 ^ab^	0.330	8.49	8.19	0.635	8.47	8.22	0.692	0.029
Gra (%)	56.32	56.93	55.08	53.45	1.095	55.70	55.19	0.825	56.63	54.27	0.311	0.626
RBC (m/mm^3^)	6.32	6.62	5.87	6.96	0.176	6.09	6.79	0.050	6.47	6.41	0.862	0.246
Hct (%)	35.02	36.32	34.98	37.98	0.999	35.00	37.15	0.312	35.67	36.48	0.698	0.686
Hb (g/dl)	11.87	12.27	11.48	14.10	0.407	11.68	13.18	0.058	12.07	12.79	0.345	0.155
THR (m/mm^3^)	329.50	325.50	325.33	378.83	19.063	327.42	352.17	0.539	327.50	352.08	0.542	0.476
**Biochemical parameters**												
ALB (g/dL)	2.63	2.57	2.42	2.52	0.061	2.53	2.54	0.897	2.60	2.47	0.305	0.518
ALT (U/L)	117.33	115.33	123.83	126.50	4.101	120.58	120.92	0.969	116.33	125.17	0.314	0.788
AST (U/L)	69.50	74.83	47.38	68.83	3.839	58.44	71.83	0.062	72.17	58.11	0.051	0.247
CHOL (mg/dL)	75.00	70.00	74.5.00	76.66	1.739	74.75	73.33	0.695	72.50	75.58	0.397	0.326
CK (U/L)	1189.50	1014.00	1017.83	1221.00	118.797	1103.67	1119.00	0.952	1101.75	1120.92	0.940	0.456
GLU (mg/dL)	92.17	98.17	100.83	92.00	4.556	96.50	95.08	0.884	95.17	96.42	0.898	0.450
TBIL (mg/dL)	0.09	0.12	0.07	0.13	0.013	0.81	0.12	0.130	0.10	0.10	1.000	0.421
TRIG (mg/dL)	49.00	48.17	63.33	54.83	2.446	56.17	51.50	0.311	48.58	59.08	0.030	0.404

Group A, control diet; Group B, diet with 100 g/kg *T. molitor* meal; Group C, diet with 0.5 g/kg chitosan; Group D, diet with 100 g/kg *T. molitor* meal and 0.5 g/kg chitosan. *N* = 6 per treatment. ^a, b^ Mean values with different superscripts are significantly (*p* ≤ 0.05) different. WBC, white blood cells; Lym, lymphocytes; Mon, monocytes; Gra, granulocytes; RBC, red blood cells; Hct, hematocrit; Hb, hemoglobin; THR, thrombomodulin. ALB, albumin; ALT, alanine aminotransferase; AST, aspartate aminotransferase; CHOL, cholesterol; CK, creatine kinase; GLU, glucose; TBIL, total bilirubin; TRIG, triglycerides.

**Table 6:** Effect of dietary addition of *Tenebrio molitor* larvae meal and chitosan on meat microbial populations of growing pigs.

Shoulder Meat Microbiota (Log_10_ CFU/g)	Treatments					Effect of *T. molitor* Meal			Effect of Chitosan			Interaction
	Group A	Group B	Group C	Group D	SEM	Without *T. molitor*	With *T. molitor*	*p*	Without Chitosan	With Chitosan	*p*	*p*
Total microbes	5.82	5.11	5.15	5.51	0.157	5.48	5.31	0.584	5.33	5.40	0.674	0.101
*Escherichia coli*	4.27	2.44	3.95	2.60	0.227	4.11	2.52	<0.001	3.36	3.27	0.780	0.413
*Clostridium* spp.	3.24	2.01	2.32	2.38	0.191	2.78	2.20	0.112	2.63	2.35	0.441	0.081
*Campylobacter jejuni*	3.44	2.31	3.13	2.47	0.162	3.29	2.39	0.004	2.88	2.80	0.738	0.390
*Staphylococcus* spp.	4.80	4.61	4.27	4.21	0.099	4.53	4.41	0.496	4.71	4.24	0.019	0.700
*Staphylococcus aureus*	2.60	2.46	1.96	2.63	0.121	2.28	2.54	0.263	2.53	2.29	0.309	0.088
**Belly meat microbiota (Log_10_ CFU/g)**												
Total microbes	6.04	6.21	5.92	6.03	0.149	5.98	6.12	0.676	6.12	5.97	0.649	0.932
*Escherichia coli*	4.31	3.37	3.62	3.17	0.212	3.96	3.27	0.110	3.84	3.39	0.290	0.560
*Clostridium* spp.	3.24	2.01	2.32	2.38	0.191	2.78	2.20	0.112	2.63	2.35	0.441	0.081
*Campylobacter jejuni*	3.41	2.98	2.84	3.25	0.155	3.12	3.12	0.982	3.19	3.05	0.651	0.213
*Staphylococcus* spp.	4.17	4.09	4.15	3.61	0.753	4.16	3.85	0.384	4.13	3.88	0.478	0.514
*Staphylococcus aureus*	2.40	2.46	2.22	1.93	0.134	2.31	2.20	0.683	2.43	2.08	0.211	0.528
**Boneless steak meat microbiota (Log_10_ CFU/g)**												
Total microbes	4.35	4.34	4.12	3.90	0.097	4.24	4.12	0.555	4.35	4.01	0.091	0.584
*Escherichia coli*	2.74	2.08	1.89	1.58	0.186	2.31	1.83	0.178	2.41	1.73	0.066	0.625
*Clostridium* spp.	1.45	1.47	1.53	1.63	0.070	1.49	1.55	0.692	1.46	1.58	0.432	0.770
*Campylobacter jejuni*	3.32	2.98	2.85	2.59	0.158	3.09	2.79	0.361	3.15	2.72	0.192	0.897
*Staphylococcus* spp.	3.09	2.32	3.07	2.47	0.173	3.08	2.40	0.059	2.71	2.77	0.848	0.811
*Staphylococcus aureus*	2.91	2.11	2.06	1.80	0.228	2.49	1.96	0.254	2.51	1.94	0.221	0.562

Group A, control diet; Group B, diet with 100 g/kg *T. molitor* meal; Group C, diet with 0.5 g/kg chitosan; Group D, diet with 100 g/kg *T. molitor* meal and 0.5 g/kg chitosan. *N* = 6 per treatment.

**Table 7:** Effect of dietary addition of *Tenebrio molitor* larvae meal and chitosan on meat chemical composition of growing pigs.

Ham Meat Chemical Composition (%)	Treatments					Effect of *T. molitor* Meal			Effect of Chitosan			Interaction
	Group A	Group B	Group C	Group D	SEM	Without *T. molitor*	With *T. molitor*	*p*	Without Chitosan	With Chitosan	*p*	*p*
Fat	2.64	3.2	2.94	3.06	0.156	2.79	3.13	0.310	2.92	3.00	0.813	0.504
Protein	19.56	20.06	19.82	20.07	0.115	19.69	20.07	0.118	19.81	19.95	0.559	0.576
Moisture	76.89	76.09	76.93	76.32	0.137	76.91	76.20	0.009	76.49	76.62	0.586	0.692
Collagen	1.02	0.89	1.11	1.03	0.038	1.07	0.96	0.153	0.95	1.07	0.133	0.715
Ash	0.98	0.97	1.03	1.04	0.026	1.01	1.00	0.958	0.98	1.03	0.322	0.791
**Boneless steak meat chemical composition (%)**												
Fat	3.18	2.57	2.75	2.98	0.121	2.96	2.77	0.434	2.87	2.86	0.967	0.097
Protein	19.80	20.61	20.34	20.47	0.140	20.07	20.54	0.094	20.21	20.41	0.463	0.210
Moisture	75.97	76.05	76.22	75.79	0.119	76.10	75.92	0.491	76.01	76.01	0.984	0.313
Collagen	1.17	1.08	1.20	1.27	0.039	1.19	1.17	0.857	1.12	1.23	0.178	0.301
Ash	1.05	0.98	0.96	0.93	0.019	1.00	0.95	0.153	1.02	0.94	0.041	0.519
**Shoulder meat chemical composition (%)**												
Fat	5.22	5.50	5.41	5.91	0.169	5.31	5.70	0.275	5.36	5.66	0.395	0.745
Protein	18.43	18.21	18.42	18.32	0.068	18.43	18.26	0.261	18.32	18.37	0.713	0.683
Moisture	75.56	75.55	75.42	75.17	0.149	75.49	75.36	0.692	75.56	75.30	0.419	0.715
Collagen	1.31	1.33	1.21	1.10	0.046	1.26	1.21	0.594	1.32	1.15	0.078	0.452
Ash	0.97	0.90	0.93	0.94	0.020	0.95	0.92	0.437	0.94	0.94	0.963	0.411
**Bellymeat chemical composition (%)**												
Fat	9.87	8.61	8.61	8.99	0.274	9.24	8.80	0.424	9.24	8.80	0.430	0.149
Protein	16.93	17.55	17.33	17.49	0.175	17.13	17.52	0.300	17.24	17.41	0.646	0.542
Moisture	72.27	72.89	73.09	72.84	0.208	72.68	72.87	0.670	72.58	72.97	0.382	0.322
Collagen	1.66	1.67	1.62	1.50	0.062	1.64	1.59	0.706	1.67	1.56	0.427	0.608
Ash	1.00	0.90	0.93	0.81	0.024	0.96	0.85	0.015	0.95	0.87	0.064	0.922

Group A, control diet; Group B, diet with 100 g/kg *T. molitor* meal; Group C, diet with 0.5 g/kg chitosan; Group D, diet with 100 g/kg *T. molitor* meal and 0.5 g/kg chitosan. *N* = 6 per treatment.

**Table 8:** Effect of dietary addition of *Tenebrio molitor* larvae meal and chitosan on meat oxidative stability, pH, and color characteristics of growing pigs.

Total Phenols (g/L)	Treatments					Effect of *T. molitor* Meal			Effect of Chitosan			Interaction
	Group A	Group B	Group C	Group D	SEM	Without *T. molitor*	With *T. molitor*	*p*	Without Chitosan	With Chitosan	*p*	*p*
Shoulder meat	1.96	5.31	4.54	5.27	0.448	3.25	5.29	0.010	3.64	4.90	0.088	0.079
Belly meat	1.83	2.04	2.36	2.38	0.088	2.09	2.21	0.463	1.94	2.37	0.013	0.535
Boneless steak meat	3.54	5.25	4.34	4.18	0.283	3.94	4.72	0.169	4.39	4.26	0.809	0.100
**MDA (ng/g)—Day 1**												
Shoulder meat	2.34	1.45	0.62	1.59	0.427	1.60	1.52	0.965	1.89	1.18	0.404	0.324
Belly meat	2.63	1.35	0.82	0.75	0.261	1.86	1.05	0.042	2.08	0.79	0.002	0.061
**MDA (ng/g)—Day 7**												
Shoulder meat	27.44 ^b^	20.16 ^a^	20.56 ^a^	20.26 ^a^	0.889	24.00	20.21	0.001	23.80	20.41	0.002	0.002
Belly meat	17.98 ^b^	12.69 ^a^	15.21 ^ab^	16.30 ^ab^	0.759	16.59	14.49	0.128	15.33	15.75	0.749	0.029
**pH**												
Shoulder meat	5.84	5.76	5.73	5.76	0.020	5.78	5.76	0.601	5.80	5.74	0.155	0.185
Belly meat	5.96	5.94	5.85	5.85	0.017	5.90	5.90	0.796	5.95	5.85	0.002	0.854
Boneless steak meat	5.95	5.87	6.02	6.08	0.032	5.99	5.98	0.867	5.91	6.05	0.032	0.252
Color L*												
Shoulder meat	63.22	61.50	58.68	57.86	0.956	60.95	59.68	0.487	62.36	58.27	0.036	0.804
Belly meat	64.40	58.64	62.94	62.52	1.042	63.67	60.58	0.143	61.52	62.73	0.555	0.202
Boneless steak meat	72.14	72.32	71.76	67.72	0.783	71.95	70.02	0.193	72.23	69.74	0.099	0.157
Color a*												
Shoulder meat	15.14	14.48	17.02	13.86	0.788	16.08	14.17	0.254	14.81	15.44	0.701	0.450
Belly meat	13.92	15.94	13.60	14.80	0.679	13.76	15.37	0.270	14.93	14.20	0.611	0.775
Boneless steak meat	8.08	7.14	5.26	6.68	0.494	6.67	6.91	0.804	7.61	5.97	0.103	0.232
Color b*												
Shoulder meat	12.32	13.24	12.94	12.00	0.364	12.63	12.62	0.990	12.78	12.47	0.687	0.236
Belly meat	10.12 ^a^	11.54 ^ab^	12.16 ^b^	11.08 ^ab^	0.320	11.14	11.31	0.776	10.83	11.62	0.198	0.050
Boneless steak meat	14.98	16.08	17.16	19.68	0.698	16.07	17.88	0.166	15.53	18.42	0.034	0.577

Group A, control diet; Group B, diet with 100 g/kg *T. molitor* meal; Group C, diet with 0.5 g/kg chitosan; Group D, diet with 100 g/kg *T. molitor* meal and 0.5 g/kg chitosan. *N* = 6 per treatment. ^a, b^ Mean values with different superscripts are significantly (*p* ≤ 0.05) different. Lightness (L*), redness (a*), and yellowness (b*) values.

**Table 9:** Effect of dietary addition of *Tenebrio molitor* larvae meal and chitosan on shoulder meat fatty acid composition of growing pigs.

Shoulder Meat Fatty Acids	Treatments					Effect of *T. molitor* Meal			Effect of Chitosan			Interaction
	Group A	Group B	Group C	Group D	SEM	Without *T. molitor*	With *T. molitor*	*p*	Without Chitosan	With Chitosan	*p*	*p*
C14:0 (Myristic)	0.30 ^b^	0.06 ^a^	0.09 ^a^	0.07 ^a^	0.032	0.19	0.07	0.002	0.18	0.08	0.010	0.004
C15:0 (Pentadecanoic)	0.29 ^b^	0.05 ^a^	0.15 ^ab^	0.13 ^a^	0.113	0.22	0.09	0.028	0.17	0.14	0.543	0.048
C15:1 (cis-10-Pentadecenoic)	2.01	1.64	0.62	0.96	2.04	1.32	1.30	0.955	1.82	0.79	0.007	0.249
C16:0 (Palmitic)	28.40	26.89	27.90	25.57	0.787	28.15	26.23	0.287	27.64	26.73	0.602	0.813
C16:1 (Palmitoleic)	0.09 ^a^	0.84 ^b^	4.02 ^d^	2.32 ^c^	0.463	2.06	1.58	0.063	0.47	3.17	<0.001	0.001
C17:0 (Heptadecanoic)	0.50	0.30	0.20	0.27	0.062	0.35	0.28	0.586	0.40	0.24	0.208	0.284
C17:1 (cis-10-Heptadecenoic)	0.53	0.82	0.26	0.39	0.071	0.40	0.61	0.029	0.68	0.33	0.002	0.356
C18:0 (Stearic)	12.43	10.49	10.93	9.29	0.459	11.68	9.89	0.039	11.46	10.11	0.100	0.843
C18:1n9t (Elaidic)	0.05	0.06	0.08	0.09	0.005	0.06	0.07	0.078	0.05	0.08	0.001	0.780
C18:1n9c (Oleic)	23.38	21.78	25.42	26.11	0.607	24.40	23.95	0.563	22.58	25.77	0.003	0.165
C18:2n6t (Linolelaidic)	0.06	0.07	0.05	0.06	0.007	0.06	0.06	0.572	0.07	0.05	0.433	0.733
C18:2n6c (Linoleic)	24.70	29.28	24.71	29.08	0.778	24.70	29.18	0.001	26.99	26.89	0.921	0.912
C18:3n6 (γ-Linolenic)	0.07	0.05	0.09	0.18	0.020	0.08	0.12	0.294	0.06	0.14	0.036	0.116
C20:0 (Arachidic)	0.66	1.05	1.29	1.33	0.092	0.98	1.19	0.076	0.85	1.31	0.002	0.131
C18:3n3 (a-Linolenic)	0.23	0.42	0.52	0.43	0.042	0.37	0.43	0.433	0.33	0.48	0.049	0.057
C20:1n9c (cis-11-Eicosenoic)	0.05	0.03	0.01	0.02	0.007	0.03	0.03	0.789	0.04	0.02	0.088	0.299
C21:0 (Heneicosanoic)	0.40	0.56	0.44	0.59	0.032	0.42	0.58	0.009	0.48	0.52	0.478	0.918
C20:2 (cis-11,14-Eicossadienoic)	0.38	0.33	0.32	0.25	0.029	0.35	0.29	0.379	0.36	0.29	0.283	0.915
C22:0 (Behenic)	5.48	5.28	2.91	2.84	0.434	4.20	4.06	0.786	5.38	2.87	0.001	0.897
Σ SFA (Total Saturated)	48.47	44.69	43.90	40.10	0.999	46.18	42.39	0.006	46.58	42.00	0.002	0.991
Σ MUFA (Total Monounsaturated)	26.10	25.17	30.42	29.89	0.793	28.26	27.53	0.449	25.64	30.15	0.001	0.831
Σ PUFA (Total Polyunsaturated)	25.44	30.15	25.69	30.01	0.792	25.56	30.08	0.001	27.79	27.85	0.951	0.843
n6 (omega 6) Fatty Acids	24.83	29.39	24.85	29.33	0.785	24.84	29.36	0.001	27.11	27.09	0.983	0.963
n3 (omega 3) Fatty Acids	0.23	0.42	0.52	0.43	0.042	0.37	0.43	0.433	0.33	0.48	0.049	0.057

Group A, control diet; Group B, diet with 100 g/kg *T. molitor* meal; Group C, diet with 0.5 g/kg chitosan; Group D, diet with 100 g/kg *T. molitor* meal and 0.5 g/kg chitosan. *N* = 6 per treatment. ^a, b, c, d^ Mean values with different superscripts are significantly (*p* ≤ 0.05) different.

## Data Availability

Data are contained within the article.
